# Shiga Toxin–Producing *Escherichia coli* Infections Associated with Hemolytic Uremic Syndrome, Italy, 1988–2000

**DOI:** 10.3201/eid0901.020266

**Published:** 2003-01

**Authors:** Alberto E. Tozzi, Alfredo Caprioli, Fabio Minelli, Alessandra Gianviti, Laura De Petris, Alberto Edefonti, Giovanni Montini, Alfonso Ferretti, Tommaso De Palo, Maurizio Gaido, Gianfranco Rizzoni, the Hemolytic Uremic Syndrome Study Group

**Affiliations:** * Istituto Superiore di Sanità, Rome, Italy; † Ospedale Pediatrico Bambino Gesù, Rome, Italy; ‡ Pediatric Clinic “De Marchi,” Milan, Italy; § University of Padua, Padua, Italy; ¶ Santobono Hospital, Naples, Italy; # Giovanni XXIII Hospital, Bari, Italy; ** Regina Margherita Hospital, Turin, Italy.

**Keywords:** Shiga toxin-producing Escherichia coli, STEC, hemolytic uremic syndrome, HUS, epidemiology, laboratory diagnosis, research

## Abstract

The mean annual incidence of hemolytic uremic syndrome in persons <15 years of age in Italy from 1988 to 2000 was 0.28 per 100,000 population. Laboratory investigations showed that Shiga toxin–producing *Escherichia coli* (STEC) infection occurred in 73.1% of patients. STEC O157 was the most common serotype, but a considerable number of cases were from infections by non-O157 STEC.

Hemolytic uremic syndrome (HUS) is the most severe and specific clinical manifestation of infections with Shiga toxin (*Stx*)–producing *Escherichia coli* (STEC), especially *E. coli* O157:H7 and other enterohemorrhagic serotypes; the incidence of HUS represents a robust index of the total incidence of these infections in a population (*1*). In Italy, HUS notification is not yet mandatory, but a nationwide surveillance system was established in 1988 and has been followed on a voluntary basis with the collaboration of the Italian Society for Pediatric Nephrology. Since then, the HUS surveillance system has been maintained to monitor the incidence of the disease, describe those affected, identify the STEC serotypes associated with HUS, and investigate possible risk factors associated with STEC infection.

## The Study

The surveillance system was based on all pediatric nephrology centers that perform dialytic treatment in Italy. HUS cases were defined as patients <15 years of age with evidence of renal failure, intravascular hemolysis, and thrombocytopenia (platelet count <100,000/mm^3^) (*2*). From May 1988 to December 2000, a total of 342 HUS cases were reported to the surveillance system. Twenty-four cases were part of two outbreaks that occurred in northern Italy in 1992 (*3*) and 1993 (*4*), which were associated with *E. coli* O111 and *E. coli* O157 infections, respectively. A third cluster of three cases associated with *E. coli* O26 infection occurred in spring 1997 in Naples, in southern Italy. No outbreak of diarrhea was detected around these clusters, and no food or environmental source of infection was identified. The mean incidence per year was 0.28/100,000 population in patients <14 years of age, with a range of 0.13/100,000 population in 1991 to 0.46/100,000 population in 2000. Since 1994, a steady increase of incidence has been observed (chi square; p=0.003). We observed the highest incidence (>0.4/100,000; children <14 years of age) in the northern part of the country; the lowest incidence was reported in the insular regions, Sicily and Sardinia. The incidence was higher in children <5 years of age (mean=0.75 cases/100,000 population; range 0.33–1.10).

The incidence observed in this study is the lowest among those reported in Europe for the same period (*5*–*10*). This finding, together with the rare occurrence of outbreaks (*3*,*4*), suggests that STEC infections are relatively uncommon in Italy. In northern regions, where most cattle farming takes place, the average annual incidence was similar to that of central European countries. The very low incidence observed in most regions of southern Italy and in the islands suggests that the Mediterranean basin is an area with a low incidence of STEC infection (*10*,*11*).

Fifty-one percent of the 342 patients were boys (age range 1 month–14 years; median age 23 months). Of 11 (3.2%) patients who died, 6 were in the acute phase of HUS. Among the 274 patients for whom the clinical information was recorded, prodromal bloody diarrhea was reported in 48%, nonbloody diarrhea in 30%, and no diarrhea in 22%. In 21% of cases, diarrhea was reported in other household members before onset of HUS in the child. In addition, diarrhea among schoolmates was reported in 7.3% of cases. These findings suggest that person-to-person transmission may play a major role in the epidemiology of sporadic HUS cases, as described in other studies (*5*,*12*).

Immediately after diagnosis of HUS, stool samples were collected from patients and household contacts when possible. Specimens were examined for the presence of free fecal *Stx* by the Vero cell assay and streaked onto MacConkey agar for STEC isolation. Serum samples were collected after diagnosis of HUS and tested for antibodies to the lipopolysaccharide (LPS) of five major STEC serogroups (O157, O26, O103, O111, and O145) by enzyme-linked immunosorbent assay as described (*3*,*13*).

Clinical specimens were collected from 249 case-patients. Stools were obtained from 228 patients and at least one serum specimen from 235 patients. The combined use of microbiologic and serologic techniques provided evidence of STEC infection in 182 (73.1%) of 249 cases examined (Table), a percentage similar to those reported in similar studies (*5*,*7*–*9*,*14*,*15*).

**Table:** Evidence of STEC infection in 249 Italian children with HUS, shown by presence of prodromal diarrhea^a^

Evidence of STEC infection	Patients with symptoms no. positive/no. examined (%)					
	Bloody diarrhea	Nonbloody diarrhea	No diarrhea	No information	Total	
STEC isolation	9/88 (10.2)	4/63 (6.3)	4/39 (10.2)	1/38 (2.6)	18/228 (7.9)	
Free fecal *Stx*	23/86 (26.7)	21/63 (33.3)	10/38 (26.3)	9/38 (23.7)	63/225 (28.0)	
Antibodies to LPS	61/88 (69.3)	39/63 (61.9)	14/41 (34.1)	30/43 (69.8)	144/235 (61.3)	
Any	73/92 (79.3)	50/67 (74.6)	22/42 (52.4)	37/48 (77.1)	182/249 (73.1)	

^a^STEC, Shiga toxin–producing *Escherichia coli*; HUS, hemolytic uremic syndrome; *Stx*, Shiga toxin; LPS, lipopolysaccharide.

We documented both microbiologic and serologic evidence of STEC infection in a considerable proportion of patients who did not have diarrhea (52.4%), although less frequently than in patients with bloody diarrhea (79.3%; chi square p=0.001) and nonbloody diarrhea (74.6%; p=0.01). A similar proportion of patients with STEC infection without diarrhea has also been described in other European studies (*7*–*9*), suggesting that Shiga toxins are able to translocate across the intestinal mucosa and reach the target endothelial cells even if the STEC infection does not result in overt diarrhea.

Despite the use of different diagnostic methods, 26.9% of patients had no evidence of STEC infection. Some patients may have had an infection caused by a STEC strain belonging to a serogroup that was not included in the LPS panel used in this study. In addition, some patients with STEC infection apparently do not have a serologic response (*7*).

Stool examination provided evidence of STEC infection in 67 (29.4%) of 228 patients examined. Free fecal *Stx* was identified in stool samples from 63 (28.0%) of 225 patients examined. Most positive samples contained *Stx*2 alone (47 samples) or in combination with *Stx*1 (11 samples). Nineteen STEC strains were isolated from 18 patients (7.9%); these strains were from serogroups O157 (6 strains), O111 (4 strains), O26 (2 strains), O55 (2 strains), O86, O113, O118, O120, and undetermined O (one strain each). The proportion of STEC recovery from stools, similar to that reported in other studies (*7*,*14*), may be low because of the interval between onset of diarrhea and stool collection (median 8 days), antimicrobial therapy administered to many patients before and after the development of HUS (data not shown), or the freezing, storing, or shipping of the specimens.

Antibodies to LPS were detected in the sera of 144 (61.3%) of 235 patients examined; in 115, this presence was the only evidence of STEC infection. Antibodies were detected to O157 (72 cases), O26 (31 cases), O111 (15 cases), O145 (15 cases), and O103 (8 cases). Three patients had antibodies to two serogroups: O157 and O26, O26 and O145, and O26 and O103.

Serotyping of isolated STEC strains and detection of serogroup-specific LPS antibodies showed evidence of infection with STEC O157 in 74 patients. Thirty-four patients had infection with STEC O26, 17 with STEC O111, 16 with O145, and 9 with O103. As shown in other studies, *E. coli* O157 was the most common STEC serogroup associated with HUS. However, >50% of STEC-positive cases had evidence of infection with non-O157 STEC. This proportion is higher than that reported in studies conducted in the United States (*14*), France (*7*), United Kingdom (*6*), Belgium (*8*), and the Netherlands (*15*) and suggests that the circulation of STEC O157 in Italy is lower than in other countries.

The distribution of the four most frequent STEC serogroups associated with HUS cases by year is shown in the Figure. Infections with non-O157 serogroups, in particular those with *E. coli* O26, increased over time and since 1996 have outnumbered those with STEC O157. Infections with STEC O111 were frequent from 1990 to 1993, after which they became rare. This observation emphasizes that the incidence of non-O157 serotypes may vary over time.

**Figure:**
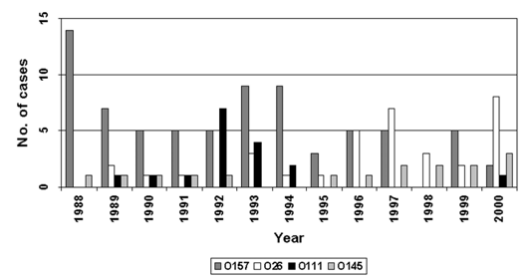
Distribution of hemolytic uremic syndrome cases associated with Shiga toxin–producing *Escherichia coli* O157, 026, O111, and O145, by year.

The frequency of STEC O157 infection increased with age; non-O157 infections were more common in younger children. This finding is in agreement with the observation that non-O157 serotypes were the leading cause of HUS in young children in Germany (*9*) and could reflect differences in the epidemiology or in the pathogenic mechanism of these infections with respect to those sustained by STEC O157.

For 115 STEC-positive HUS cases, we obtained stool specimens from 333 household contacts and 119 household contacts of 36 STEC-negative HUS cases. Laboratory evidence of STEC infection was found in 10 (3.3%) and in 1 (0.8%) of household contacts of STEC-positive and STEC-negative HUS cases, respectively. Two household contacts were infected with STEC strains belonging to a serogroup different from that associated with the HUS case. One of 11 STEC-positive household contacts reported diarrhea. Our findings underscore the potential for person-to-person transmission of STEC infection.

## Conclusions

The results of this 13-year surveillance indicate that the overall incidence of HUS in Italy is lower than in other European countries, although a small but steady increase has been observed since 1994. As in most countries, STEC O157 represented the most common serotype, but a considerable proportion of cases was due to infections by non-O157 STEC, particularly O26, increasing over the last 5 years. Surveillance of HUS can provide useful information about the trend of STEC infection in the general population and represents an important means to identify STEC serotypes that are highly pathogenic to humans and may emerge as public health threats.

## References

[1] Mahon BE, Griffin PM, Mead PS, Tauxe RV. Hemolytic-uremic syndrome surveillance to monitor trends in infection with *Escherichia coli* O157:H7 and other Shiga toxin-producing *E. coli.* Emerg Infect Dis. 1997;3:409–12. 10.3201/eid0303.970329 9284395 PMC2627651

[2] Gianviti A, Rosmini F, Caprioli A, Corona R, Matteucci MC, Principato F, Haemolytic-uraemic syndrome in childhood: surveillance and case-control studies in Italy. Pediatr Nephrol. 1994;8:705–9. 10.1007/BF00869095 7696109

[3] Caprioli A, Luzzi I, Rosmini F, Resti C, Edefonti A, Perfumo F, Communitywide outbreak of hemolytic-uremic syndrome associated with non-0157 verocytotoxin-producing *Escherichia coli.* J Infect Dis. 1994;169:208–11. 8277184 10.1093/infdis/169.1.208

[4] Tozzi AE, Niccolini A, Caprioli A, Luzzi I, Montini G, Zacchello G, A community outbreak of haemolytic-uraemic syndrome in children occurring in a large area of northern Italy over a period of several months. Epidemiol Infect. 1994;113:209–20. 10.1017/S0950268800051645 7925660 PMC2271537

[5] Bitzan M, Ludwig K, Klemt M, Konig H, Buren J, Muller-Wiefel DE. The role of *Escherichia coli* 0157 infections in the classical (enteropathic) haemolytic uraemic syndrome: results of a central european, multicentre study. Epidemiol Infect. 1993;110:183–96. 10.1017/S0950268800068102 8472763 PMC2272264

[6] Milford DV, Taylor CM, Guttridge B, Hall SM, Rowe B, Kleanthous H. Haemolytic uraemic syndromes in the British Isles 1985–8: association with verocytotoxin producing *Escherichia coli*. Part 1: clinical and epidemiological aspects. Arch Dis Child. 1990;65:716–21. 10.1136/adc.65.7.716 2201261 PMC1792437

[7] Decludt B, Bouvet P, Mariani-Kurkdjian P, Grimont F, Grimont PAD, Hubert B, Haemolytic uraemic syndrome and Shiga toxin-producing *Escherichia coli* infection in children in France. Epidemiol Infect. 2000;124:215–20. 10.1017/S0950268899003623 10813145 PMC2810903

[8] Piérard D, Cornu D, Proesmans W, Dediste A, Jacobs F, Van de Walle J, Hemolytic uremic syndrome in Belgium: incidence and association with verocytotoxin-producing *Escherichia coli* infection. Clin Microbiol Infect. 1999;5:16–22. 10.1111/j.1469-0691.1999.tb00093.x 11856208

[9] Verweyen HM, Karch H, Allerberger F, Zimmerhackl B. Enterohemorragic *E. coli* in pediatric HUS: a prospective study in Germany and Austria. Infection. 1999;27:341–7. 10.1007/s150100050040 10624594

[10] Caprioli A, Tozzi AE. Epidemiology of Shiga-toxin–producing *Escherichia coli* infections in continental Europe. In: Kaper JB, O’Brien A, editors. *Escherichia coli* O157:H7 and other Shiga-toxin–producing *E.coli*. Washington: American Society for Microbiology, 1998; p. 38–48.

[11] Tozzi AE, Gorietti S, Caprioli A. Epidemiology of human infections by *Escherichia coli* O157 and other verocytotoxin-producing *E. coli*. In: Duffy G, Garvey P, McDowell DA, editors. Verocytotoxigenic *Escherichia coli*. Food & Nutrition Press, Inc. 2001; p. 161–79.

[12] Rowe PC, Orrbine E, Lior H, Wells GA, McLaine PN. Diarrhoea in close contacts as a risk factor for childhood haemolytic uraemic syndrome: the CPKDRC co-investigators. Epidemiol Infect. 1993;110:9–16. 10.1017/S0950268800050627 8432328 PMC2271967

[13] Caprioli A, Luzzi I, Rosmini F, Pasquini P, Cirrincione R, Gianviti A, Hemolytic-uremic syndrome and verocytotoxin-producing *Escherichia coli* infection in Italy. J Infect Dis. 1992;166:154–8. 1607689 10.1093/infdis/166.1.154

[14] Banatvala N, Griffin PM, Greene KD, Barrett TJ, Bibb WF, Green JH, The United States national prospective hemolytic uremic syndrome study: microbiologic, serologic, clinical, and epidemiologic findings. J Infect Dis. 2001;183:1063–70. 10.1086/319269 11237831

[15] Van de Kar NCAJ, Roelofs HGR, Muytjens HL, Tolboom JJM, Roth B, Proesmans W, Verocytotoxin-producing *Escherichia coli* infection in hemolytic uremic syndrome in part or Western Europe. Eur J Pediatr. 1996;155:592–5. 10.1007/s004310050448 8831084

